# An update on the mechanisms and risk factors for anesthesia-related cardiac arrest in children: a narrative review

**DOI:** 10.1016/j.bjane.2024.844519

**Published:** 2024-05-27

**Authors:** Leandro Gobbo Braz, Jose Reinaldo Cerqueira Braz, Teofilo Augusto Araújo Tiradentes, Daniela de Sa Menezes Porto, Cristiano Martins Beserra, Luiz Antonio Vane, Paulo do Nascimento Junior, Norma Sueli Pinheiro Modolo, Mariana Gobbo Braz

**Affiliations:** Universidade Estadual Paulista (UNESP), Faculdade de Medicina de Botucatu, Departamento de Especialidades Cirúrgicas e Anestesiologia, Comissão de Estudos de Parada Cardíaca e Mortalidade em Anestesia, Botucatu, SP, Brazil

**Keywords:** Cardiac arrest, Children, Developed countries, Low-income countries

## Abstract

The relation between surgery and anesthesia safety in children and a country's Human Development Index (HDI) value has been described previously. The aim of this narrative review was to provide an update on the mechanisms and risk factors of Anesthesia-Related Cardiac Arrest (ARCA) in pediatric surgical patients in countries with different HDI values and over time (pre-2001 vs. 2001‒2024). Electronic databases were searched up to March 2024 for studies reporting ARCA events in children. HDI values range from 0 to 1 (very-high-HDI countries: ≥ 0.800, high-HDI countries: 0.700‒0.799, medium-HDI countries: 0.550‒0.699, and low-HDI countries: < 0.550). Independent of time, the proportion of children who suffered perioperative Cardiac Arrest (CA) attributed to anesthesia-related causes was higher in very-high-HDI countries (50%) than in countries with HDI values less than 0.8 (15‒36%), but ARCA rates were higher in countries with HDI values less than 0.8 than in very-high-HDI countries. Regardless of the HDI value, medication-related factors were the most common mechanism causing ARCA before 2001, while cardiovascular-related factors, mainly hypovolemia, and respiratory-related factors, including difficulty maintaining patent airways and adequate ventilation, were the major mechanisms in the present century. Independent of HDI value and time, a higher number of ARCA events occurred in children with heart disease and/or a history of cardiac surgery, those aged younger than one year, those with ASA physical status III‒V, and those who underwent emergency surgery. Many ARCA events were determined to be preventable. The implementation of specialized pediatric anesthesiology and training programs is crucial for anesthesia safety in children.

## Introduction

Perioperative Cardiac Arrest (CA) remains one of the most catastrophic complications of pediatric anesthesia. The occurrence of perioperative CA in children is related not only to the patient's condition/disease but also to factors such as surgical management and inappropriate anesthesia.^1^, ^2^, ^3^

The Human Development Index (HDI), which was established by the United Nations Development Programme, is calculated based on rates of enrollment in higher education and literacy, per capita income, and life expectancy.^4^ The HDI value ranges from 0 to 1, representing the lowest and highest levels of development, respectively. The relationship between surgery and anesthesia safety and the HDI value has been described in previous studies involving children,^5^ older patients^6^ and patients of all ages.^7^, ^8^, ^9^, ^10^

A recent systematic review of global studies involving children demonstrated that Anesthesia-Related Cardiac Arrest (ARCA) rates were inversely correlated with HDI values and did not change over time in either high-HDI countries (HDI value ≥ 0.8) or low-HDI countries (HDI value < 0.8); however, ARCA rates were significantly higher in low-HDI countries than in high-HDI countries and higher in children aged less than one year than in children aged 1 year or older in high-HDI and low-HDI countries.^11^

Investigations into the causes and factors associated with ARCA in children have been conducted for more than 150 years^12^^,^^13^ but have been limited by the low incidence and lack of consistent definitions of such events.^14^

Owing to considerable advances in safe anesthesia care for children in recent decades, especially in high-HDI countries, and the gap in health care systems between low- and high-HDI countries,^7^, ^8^, ^9^, ^10^ we verified whether the ARCA mechanisms and risk factors in pediatric surgical patients differ in countries with different HDIs and over time.

## Methods

We constructed our narrative review according to the scale for the quality assessment of narrative review articles.^15^ Ethical approval was not necessary because we conducted a narrative review.

We searched the literature to identify all studies reporting perioperative CA and/or ARCA in a surgical child population. We searched the PubMed, Embase, and Lilacs databases from inception to March 27, 2024. The search was conducted using index terms (e.g., MeSH) and text words, as well as word variants for “an(a)esthesia”, “cardiac arrest”, and “mortality”, including a list of synonyms. Titles and abstracts were screened, and the full texts of studies deemed potentially relevant were reviewed. No language restrictions were imposed, and translation services were used when necessary. We included only published full-text articles. Stand-alone abstracts and unpublished studies were excluded from the review.

The outcomes of interest were the mechanisms and risk factors for ARCA events in children in countries with different HDIs. ARCA events are subdivided entirely and partially. The entire ARCA subgroup included events exclusively attributed to anesthesia personnel or the anesthetic process (e.g., ventilatory depression with resultant hypoxemic CA after intravenous opioid injection in a stable child patient without comorbidities). The partial ARCA subgroup included events in which anesthesia personnel, or the anesthetic process clearly played a role, but other factors related to the patient's disease/condition or surgical procedure may have also played a role in the sequence of events reported (e.g., CA immediately after the induction of anesthesia in an unstable, hypovolemic child patient). ARCA events were classified by the authors of the studies included in this review. Many of the included studies separated the results into entire and partial ARCA subgroups. For this analysis, we summed both entire and partial ARCA events.

Considering the differences in surgery and anesthesia safety among countries, as demonstrated in studies,^16^^,^^17^ the country development level was classified according to the HDI established by the United Nations Development Programme as follows: ≥ 0.800 for very-high-HDI countries, 0.700‒0.799 for high-HDI countries, 0.550‒0.699 for medium-HDI countries, and < 0.550 for low-HDI countries.^4^ Since the HDI of a country may change over time and studies often report data spanning several years, the HDI of each country was calculated as the mean of the HDIs in the first and last years studied.^10^^,^^18^

Considering that the focus of this study was on identifying changes in the mechanisms and causes of ARCA in children in the current century in relation to those in the last century, we divided the study into two periods: pre-2001 (last century) and 2001‒2022 (present century).

**Figure 1:**
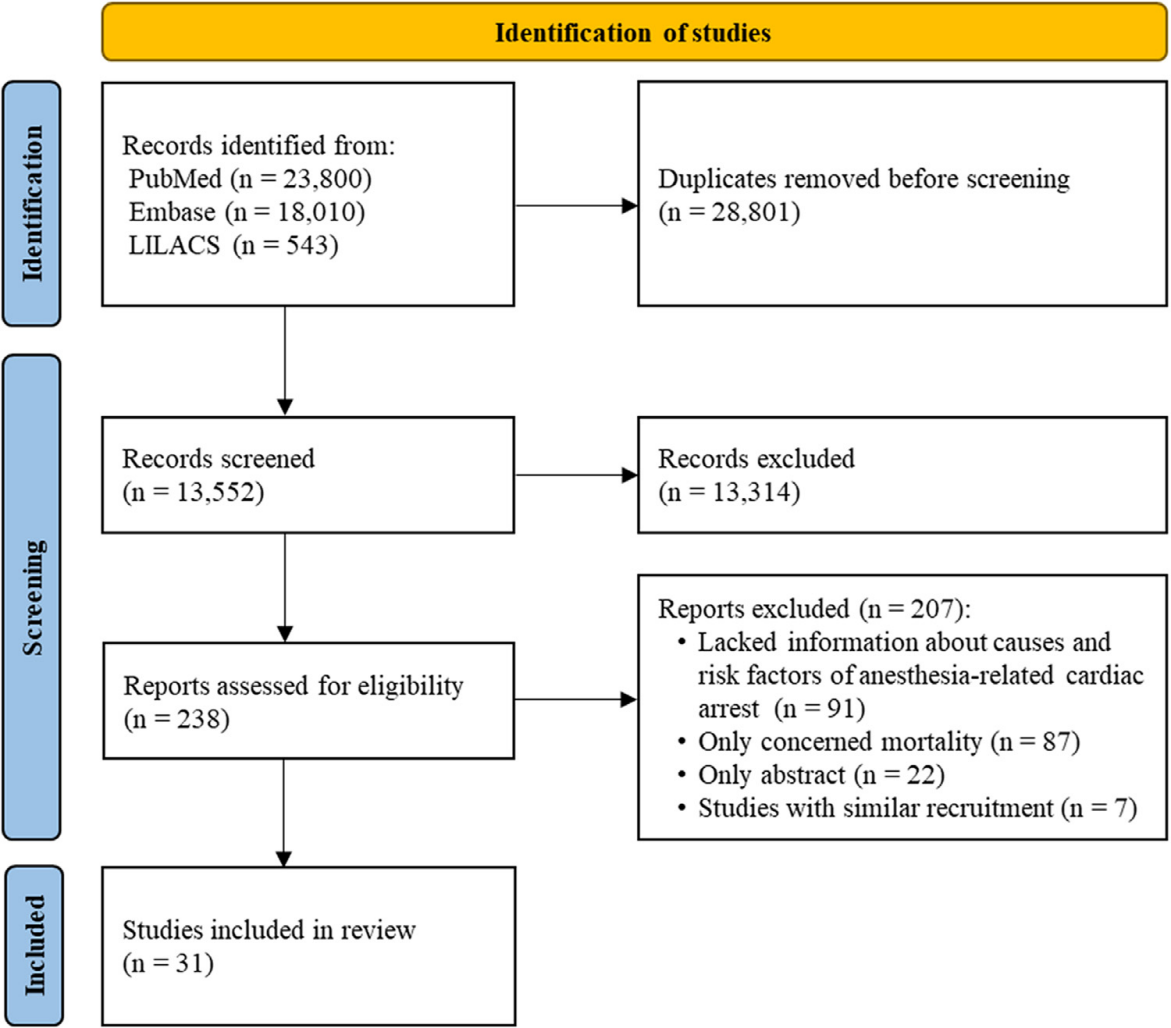
Flowchart of study identification.

Our search strategy identified 42,353 citations. After excluding 28,801 duplicates, the titles and abstracts of remaining articles were reviewed, and an additional 13,314 irrelevant studies were excluded. We retrieved 238 potentially relevant full-text articles for detailed evaluation. Overall, 31 studies met the predefined outcomes of interest (Fig. 1). The characteristics of the included studies are reported in Supplementary Table 1.

### Perioperative CA attributed to anesthesia-related causes in children

Two studies from a very-high-HDI country (USA) demonstrated high proportions of children with perioperative CA attributed to anesthesia-related causes in different periods: 52% in 1994‒1997^19^ and 49% in 1998‒2004.^20^ Two studies in children from a very-high-HDI country (Germany) in the present century reported similar findings, with ARCA events accounting for 50% of the perioperative CA events and non-ARCA events accounting for the remaining 50% of cases in 2008‒2016^21^ and 2008‒2018.^22^

In contrast, studies from a high-HDI country (Brazil) in the last century^23^ and the present century^3^ demonstrated lower proportions of children with perioperative CA events attributed to anesthesia-related causes (20% in 1996‒2004 and 15% in 2005‒2010, respectively), while disease/condition-related CA was the most important factor triggering perioperative CA, with proportions of 80% and 73%, respectively. Studies from a medium-HDI country (India)^2^ and a low-HDI country (Nigeria)^24^ conducted in the present century also demonstrated lower proportions of children with perioperative CA attributed to ARCA events than non-ARCA events (26% and 36%, respectively, versus 74% and 64%, respectively). Interestingly, a study from a high-HDI country (Thailand) in the present century reported an increase in the proportion of perioperative CA attributed to anesthesia-related causes in different periods, from 33% in 2003‒2004^25^ to 54% in 2014‒2019.^26^

According to the authors, the different proportions of children with ARCA events attributed to perioperative CA among countries with different HDIs were due to the high proportions of disease/condition-related CA as a factor triggering perioperative CA in countries with an HDI value less than 0.8.^2^^,^^3^^,^^23^, ^24^, ^25^, ^26^ Many of these children live outside the centers where they are definitively managed, and considering the reported poor index of suspected health disorders among health care providers in primary and secondary care facilities and the insufficient number of surgical beds and neonatal and/or pediatric intensive care units, there is a delay in initial diagnosis and referral to appropriate centers, resulting in delayed diagnosis and intervention.^27^

### Mechanisms of ARCA events in children

Reports prior to 2001 demonstrated that medication-related factors were the most common mechanism of ARCA events in children. Thus, a pioneering study in a very-high-HDI country (USA)^13^ demonstrated a high proportion of ARCA events due to cardiovascular depression caused by inhalation anesthetic overdose (ether or chloroform) followed by, to a lesser extent, respiratory events due to hypoxia and even anoxia. The initial findings of the Perioperative Cardiac Arrest (POCA) registry in very-high-HDI countries (USA and Canada) from 1994‒1997 showed that medication-related events due to inhalation agents, especially halothane, were the most common mechanism of ARCA (37%), particularly in children with American Society of Anesthesiologists (ASA) physical status I‒II and children younger than 1 year of age, followed, to a lesser extent, by cardiovascular (32%), respiratory (20%) and equipment-related (7%) mechanisms.^19^ However, in the same study, in children with ASA physical status III‒IV, cardiovascular etiologies were the most common mechanism of ARCA. Similarly, other studies from a very-high-HDI country (USA)^28^, ^29^, ^30^ and a study from a low-HDI country (Pakistan)^1^ also showed that medication-related factors were the most common mechanism of ARCA pre-2001. Late data from the POCA registry in the 1998‒2004 period, compared to the earlier period (1994‒1997), demonstrated that the rate of ARCA events due to medication-related mechanisms declined to 18%, while ARCA events due to cardiovascular or respiratory mechanisms were more common (41% and 27%, respectively).^20^

Certainly, the discontinuation of halothane as the primary inhaled anesthetic and the introduction of new anesthetics, such as inhaled halogenated sevoflurane and intravenous propofol, contributed significantly to the decrease in medication-related ARCA events in children.^20^ Sevoflurane has been reported to have less potential for causing bradycardia^31^ and myocardial depression^32^^,^^33^ in children than halothane. Halothane also causes a higher number of hypotensive episodes than sevoflurane in children with congenital heart disease.^34^

Studies in the present century from very-high-HDI countries (Spain,^35^ South Korea,^36^ USA,^37^ and Germany^21^) demonstrated that cardiovascular-related factors and respiratory-related factors were the major mechanisms of ARCA in children, followed by medication-related factors to a lesser extent. However, studies in the same period showed that ARCA caused by respiratory-related factors occurred in greater proportion than other mechanisms in a very-high-HDI country (USA),^22^^,^^38^ a high-HDI country (Brazil),^3^ a medium-HDI country (India),^2^ and a low-HDI country (Nigeria).^39^ However, a study from a low-HDI country in Africa (Nigeria)^24^ showed that medication-related factors are still an important contributors to ARCA events due to the use of halothane as an inhaled anesthetic.

### Mechanisms of ARCA events according to the phase of anesthesia care

Studies have shown different mechanisms of ARCA events according to the phase of anesthetic care. Thus, independent of time and HDI, most of the ARCA events due to respiratory-related factors occurred in the induction phase followed by the emergence and recovery phases in very-high-HDI countries,^20^^,^^21^^,^^22^^,^^30^^,^^36^, ^37^, ^38^^,^^40^, ^41^, ^42^ high-HDI countries,^3^^,^^23^^,^^25^ a medium-HDI country^2^ and low-HDI countries,^24^^,^^39^ while most of the ARCA events due to cardiovascular-related factors occurred during anesthesia maintenance in very-high-HDI countries,^19^, ^20^, ^21^^,^^35^, ^36^, ^37^, ^38^^,^^43^ a medium-HDI country,^2^ and a low-HDI country.^44^

### Mechanisms of ARCA events according to the surgical procedure

Studies after 2000 demonstrated a significant correlation between the surgical procedure and the mechanism of ARCA events. Thus, a study from a very-high-HDI country (USA) showed that cardiovascular-related factors were the most important mechanism of ARCA events in children undergoing neurosurgery or spine surgery (71%) and cardiovascular procedures (45%), while respiratory-related factors were the most common mechanism of ARCA events in children undergoing airway, ear, nose, and throat surgeries (49%).^20^ Another study from a very-high-HDI country (Spain) also demonstrated that cardiovascular-related factors were the most important mechanism (50%) of ARCA events in children undergoing cardiovascular procedures, while respiratory-related factors were the most common mechanism of ARCA events in children undergoing noncardiovascular procedures.^35^

### Causes of ARCA events due to cardiovascular-related factors

Studies from very-high-HDI countries in the 2001‒2022 period demonstrated that the most common cause of ARCA events was hypovolemia related to blood loss^20^^,^^35^ due to the following cardiovascular-related factors:^20^ the underestimation of blood loss, lack of a central venous catheter, inadequate peripheral venous access, lack or malfunctioning of an arterial catheter, and underestimation of preexisting hypovolemia, among other causes. Other commonly cited cardiovascular causes were arrythmia, electrolyte imbalance and inadequate/inappropriate fluid therapy.^20^^,^^37^

### Causes of ARCA events due to respiratory-related factors

In the past and present centuries, with no differences in HDI values and periods, the most cited causes of respiratory events responsible for ARCA were airway obstruction mainly due to laryngospasm and bronchospasm, the inability to oxygenate or ventilate, inadvertent or premature tracheal extubation, esophageal or endobronchial intubation, aspiration and pneumothorax in very-high-HDI countries,^19^, ^20^, ^21^, ^22^^,^^30^^,^^36^, ^37^, ^38^^,^^41^^,^^42^^,^^45^^,^^46^ high-HDI countries,^3^^,^^23^^,^^25^ a medium-HDI country,^2^ and low-HDI countries.^24^^,^^39^

Children, especially neonates and infants, are at increased risk of hypoxemia because of their smaller functional residual capacity, increased heart rate, and increased metabolic requirements compared with adults; additionally, the proportionally large head and tongue, restricted submandibular space, high location of the larynx in the neck, and flaccid epiglottis in children present special challenges in airway management.^47^ Recent studies have demonstrated two major advances regarding this issue: the use of videolaryngoscopes, which has improved the first-attempt success rate of tracheal intubation and decreased the rate of airway complications,^48^ and apneic oxygenation, which has been shown to significantly prolong the safe apnea time until desaturation in neonates and infants and depends on adequate preoxygenation and the implementation of high-flow nasal oxygen during endotracheal intubation, ensuring adequate oxygenation during prolonged airway manipulation or difficult intubation.^49^

### Causes of ARCA due to medication-related or equipment-related factors

Studies from the 2001‒2022 period in very-high-HDI countries demonstrated that the main causes of ARCA in children were inhaled halogenated anesthetics, intravenous agents, local anesthetics, and allergic reactions, while the main causes of equipment-related CA were central venous catheter complications (arrythmia, pneumothorax, hemothorax, and hemopericardium), kinked or plugged tracheal tubes, and breathing circuit problems.^20^^,^^35^^,^^37^

### ARCA risk factors in children

There are many risk factors for ARCA in children, such as postnatal age, ASA physical status, heart disease, and the nature of surgery (Table 1).

**Table 1:** Risk factors of anesthesia-related cardiac arrest in children.

• Age younger than one year old, especially preterm neonates with low and very low birth weights
• ASA physical status III‒V
• Heart disease in cardiac or noncardiac surgery
• Emergency surgery
• Anesthesiologist without subspecialty pediatric training

#### Postnatal age groups

A recent review of global studies involving children demonstrated that the rate of ARCA in children has not declined over the past 60 years and is inversely related to the HDI value.^11^ This same review demonstrated that the worldwide rates of ARCA in high-HDI countries (HDI values ≥ 0.8) were 3.1-fold higher among children younger than one year old than among those aged one year or older before 2001 (5.54 vs. 1.76 per 10,000 anesthetic procedures) and 8-fold higher in 2001‒2022 (10.69 vs. 1.48 per 10,000 anesthetic procedures), while in low-HDI countries (HDI values < 0.8), these rates were 6.6-fold higher among children younger than one year old than among those ≥ 1 year of age before 2001 (10.46 vs. 1.67 per 10,000 anesthetic procedures) and 7.3-fold higher 2001‒2022 (36.02 vs. 2.86 per 10,000 anesthetic procedures).^11^ According to the authors of the review, the high ARCA rate in children younger than one year old, even in very-high-HDI countries, may be due to the increase in the number of surgical patients with a poorer ASA physical status (≥ III), as demonstrated in a global systematic review of studies.^7^ A study from a high-HDI country reported that the total proportion of children aged less than 1 year and the proportion of children aged less than one year with an ASA physical status of III to V were higher in the period from 2014 to 2016 than in the period from 2008 to 2013, confirming increases in underlying risks and complexity among pediatric surgical patients.^21^ In addition, studies have indicated increases in the numbers of preterm neonates with low and very low birth weights who are treated with surgical interventions, which are associated with high morbidity and mortality rates.^22^^,^^50^

These findings demonstrate a persistent need to improve the quantity and quality of resource utilization by increasing the number of pediatric anesthesia staff, organizing multidisciplinary discussions of adverse events, and adopting perioperative medical practices with demonstrable effectiveness, particularly in neonates and infants.

#### ASA physical status

A study from a very-high-HDI country (France)^41^ before 2001 reported a greater number of ARCA events in children with ASA physical status I‒II than in those with ASA physical status III‒V. In contrast, in the present century, studies have identified a higher risk of ARCA events in children with ASA physical status ≥ III than in those with ASA physical status I‒II in very-high-HDI countries (USA,^20^^,^^37^^,^^38^ Spain,^35^ and Germany^21^), a high-HDI country (Thailand)^26^ and a low-HDI country (Pakistan).^44^

A study of the POCA registry revealed fewer children with ASA physical status I who experienced ARCA events (7%) in the 1998‒2004 period than in the 1994‒1997 period (15%).^20^ The authors of this study also verified that 75% of the ARCA events occurred in children with ASA physical status III‒V. However, ARCA events still occurred in children with ASA physical status I in the 2001‒2022 period, as demonstrated in reports from high- and low-HDI countries^39^^,^^51^ and very-high-HDI countries.^20^^,^^37^^,^^52^

Studies from very-high-HDI countries after 2000 have demonstrated that ASA physical status III‒V is strongly associated with high ARCA rates in children according to univariable and multivariable analyses.^20^^,^^21^^,^^37^^,^^38^

#### Heart disease and/or cardiac surgery

In the present century, studies from very-high-HDI countries (USA^38^^,^^53^ and Spain^35^) demonstrated higher ARCA rates among children undergoing cardiac surgeries than among those undergoing noncardiac surgeries. It has been shown that children with heart disease have an increased frequency of ARCA events when undergoing cardiac surgery^53^^,^^54^ and noncardiac surgery,^51^^,^^53^ with a higher risk among neonates and infants; however, ARCA events were not associated with an increase in anesthesia-related mortality.^54^ Children with a single ventricle, aortic stenosis, or cardiomyopathy have an increased frequency of ARCA and mortality events.^53^

#### Emergency surgery

A study from the POCA registry in very-high-HDI countries (USA and Canada) in 1994‒1997 demonstrated that emergency surgery was a predictor of ARCA and mortality in children by multivariate analysis, with an odds ratio of 3.88 compared with that of elective surgery.^19^

Univariate analyses of several studies from very-high-HDI countries in the present century revealed a significantly higher rate of ARCA events in children undergoing emergency surgeries than in children undergoing nonemergency surgeries.^21^^,^^35^^,^^38^ However, after adjustment for an ASA physical status ≥ III and an age ≤ 6 months, a study demonstrated no significant correlation between ARCA events and emergency surgery.^38^

#### Anesthesiologist-related factors and the risk of ARCA events

A study from a very-high-HDI country (USA) in children undergoing surgeries before 2001 verified no ARCA events in the presence of pediatric anesthesiologists (0 per 10,000 anesthetic procedures), while there were four ARCA events (19.7 per 10,000 anesthetic procedures) in the presence of anesthesiologists without subspecialty pediatric training.^29^ Similarly, a study^37^ from a very-high-HDI country (USA) in 2001‒2022 demonstrated via univariate analyses that the risk of ARCA events was significantly higher for anesthesiologists with a lower caseload and/or a lower number of annual days in which anesthetic procedures were performed. In addition, these authors demonstrated that anesthesiologists with the highest academic rank and who had years of experience had higher odds ratios of experiencing ARCA events. However, after adjustment for an ASA physical status ≥ III and an age ≤ 6 months, the authors verified that the association of ARCA events with a lower number of annual days providing anesthetics remained, but the other practitioner-related factors were no longer significant. However, other studies in 2001‒2022 from very-high-HDI countries (USA and Germany) demonstrated opposite findings. Christensen and colleagues^38^ verified no significant correlation between ARCA events and pediatric anesthesiologist-related risk factors (academic rank, years of experience, annual caseload, annual days providing anesthetics, annual number of child cases, or annual number of patients with ASA physical status ≥ III, even after adjusting for an age ≤ 180 days and ASA physical status > II) in univariate or adjusted analyses. In contrast, Hohn and colleagues^21^ verified that the implementation of a specialized pediatric anesthesia team and training program in 2014 was associated with lower rates of pediatric perioperative CA (43.2% lower) and ARCA events (62.8% lower) in 2014‒2016 than in 2008‒2013.

### Timing of the ARCA events according to the phase of anesthesia care

The timing of the ARCA events varied by phase of anesthesia care regardless of the HDI value in the present century. Between 40% and 50% of the ARCA events occurred during anesthesia maintenance, 30% to 40% occurred in the anesthesia induction or tracheal intubation phases, 10% to 15% occurred during anesthesia emergence, transport, or recovery, and 5% occurred in the postoperative period.^20^^,^^25^^,^^35^^,^^37^^,^^38^

### ARCA events in children with heart disease according to the anesthetic setting

A report from a very-high-HDI country (USA) in the present century demonstrated a higher ARCA rate (21.88 per 10,000 anesthetic procedures) among children with heart disease in cardiac operating rooms than among those in radiological suites and general operating rooms according to univariate analysis (2.73 and 1.78 per 10,000 anesthetic procedures, respectively).^37^ Despite the lower proportion of ARCA events in children, a higher proportion of ARCA events with mortality occurred in nonoperating rooms than in operating rooms.^35^ As interventional techniques become more complex, more children are receiving anesthetic care outside traditional operating room settings. Challenges related to patient safety in these locations include a remote location, a limited and ergonomically inefficient anesthesia workspace, poor lighting (because of the need for fluoroscopy), different procedures and equipment, and a lack of regular interactions with personnel in these areas.^55^

### Risk of ARCA events according to the time of day and day of the week

Univariate associations between the ARCA rate and time of day (daytime vs. nighttime) and day of week (weekday vs. weekend) among children undergoing surgeries showed no significant correlation in studies from a very-high-HDI country (USA) conducted in the present century.^37^^,^^38^

### Implications of the available evidence

The use of safety monitors for oxygen, carbon dioxide and anesthetic gas administration, equipment for resuscitation, modern anesthesia equipment, and new anesthetics, as well as neonatal and pediatric intensive care units, is not routine in several high- and medium-HDI countries, especially in low-HDI countries.^17^^,^^24^^,^^56^ In low-HDI countries, the combination of poverty and poor healthcare and the increase in the child population,^2^^,^^57^ the increasing number of pediatric patients with poorer physical conditions,^17^^,^^21^ the limited anesthesia and surgical workforce^58^ and the limited number of surgical beds^57^ in conjunction with poor infection and hemorrhage control have contributed to the high perioperative CA risk in children.^2^^,^^3^^,^^23^ Thus, many ARCA events were considered preventable.^1^^,^^21^

The professional responsibility of anesthesiologists to avoid ARCA events in pediatric patients receiving anesthesia is achieved by delivering care to the best of their knowledge and ability in the preoperative, operative and postoperative conditions in conjunction with coordination of care, organizational culture, communication with nursing and surgical teams, and practice guidelines.^59^ Pediatric anesthesia in very-high-HDI countries is frequently administered by a trained anesthesiologist.^60^ However, in high- and medium-HDI countries, pediatric anesthesiologists are rare, and anesthesiologists with little experience in pediatric anesthesia often provide anesthesia services to children. In low-HDI countries, nonphysicians are often involved in providing pediatric anesthesia services due to the lack of anesthesiologists, especially in African countries.^58^

In 2015, the Safetots.org initiative (www.safetots.org) addressed the well-known perioperative risks in young children and provided a framework for the safe administration of pediatric anesthesia.^61^ Thus, the 10-Ns of anesthesia were introduced as criteria for appropriately performed pediatric anesthesia (Table 2).^61^

**Table 2:** The 10-Ns of maintenance of physiological homeostasis for the safe conduct of anesthesia in children.^61^

1. No fear
2. Normovolemia
3. Normotension
4. Normal heart rate
5. Normoxemia
6. Normocapnia
7. Normonatremia
8. Normoglycemia
9. Normothermia
10. No pain

### Limitations of this review

Some limitations of the data in this review may have affected the findings. First, considering the small number of children in many studies, especially in countries with lower HDIs,^1^, ^2^, ^3^^,^^23^^,^^24^ and because ARCA is an infrequent event, slight fluctuations in the numbers of children might have affected the reporting of these events in different studies, especially retrospective studies. Second, the small number of ARCA events in several studies, mainly from high-, medium- and low-HDI countries, restricted the review and analysis of the mechanisms of and risk factors for ARCA in children to studies from very-high-HDI countries. Third, the selective reporting bias of ARCA events may have varied according to the medico-legal circumstances of each country. Thus, ARCA events may be associated with malpractice issues; underreporting is likely in this situation. To minimize the risk of underreporting of ARCA events, the reporting of these events needs to be mandatory, and summaries need to be prepared without assigning responsibility for the CA event.^43^ Each summary was submitted anonymously to an independent study commission for analysis. Commission members from outside the institution could provide a more unbiased evaluation of these cases than faculty members from the institution of the study.^43^ Fourth, the majority of the included studies in our review are representative of experience from university hospitals and may not represent the entire spectrum of surgery and anesthesia care practices in each country. Fifth, considering that the studies included in the current review were not designed to evaluate causality, we can only infer a relationship between differences in HDI levels depending on the country and the mechanisms and risk factors for ARCA events.

## Conclusions

There were differences and similarities in the mechanisms and risk factors for ARCA events in pediatric surgical patients according to period and country HDI level. Independent of the period, the proportion of ARCA in patients experiencing perioperative CA was higher in very-high-HDI countries than in countries with lower HDI levels, but the ARCA rate was lower in very-high-HDI countries than in countries with lower HDI levels. Independent of country-HDI level, medication-related factors were the most common mechanism of ARCA before 2001, while cardiovascular-related and respiratory-related factors were the major mechanisms of ARCA in 2001‒2022. Independent of the period and country HDI value, most cardiovascular-related ARCA events occurred during anesthesia maintenance in cardiovascular surgeries, mainly due to hypovolemia, while most respiratory-related ARCA events occurred during anesthesia induction followed by the emergence and recovery phases, mainly in noncardiovascular surgeries, due to difficulty in maintaining patent airways and adequate ventilation. ARCA events at all HDI levels were higher in children with heart disease and/or who were undergoing cardiac surgery, who were younger than one year old versus one year old or older, who had an ASA physical status III‒V, who underwent emergency surgery. The implementation of specialized pediatric anesthesiologists and training programs is crucial for the safety of anesthesia in children.

## CRediT authorship contribution statement

**Leandro Gobbo Braz:** Conceptualization, Project administration, Investigation, Methodology, Writing – original draft, Writing – review & editing, Funding acquisition, Supervision. **Jose Reinaldo Cerqueira Braz:** Conceptualization, Project administration, Investigation, Methodology, Writing – original draft, Writing – review & editing, Supervision. **Teofilo Augusto Araújo Tiradentes:** Investigation, Methodology, Writing – review & editing. **Daniela de Sa Menezes Porto:** Methodology, Writing – review & editing. **Cristiano Martins Beserra:** Investigation, Writing – review & editing. **Luiz Antonio Vane:** Investigation, Writing – review & editing. **Paulo do Nascimento Junior:** Investigation, Writing – review & editing. **Norma Sueli Pinheiro Modolo:** Investigation, Writing – review & editing. **Mariana Gobbo Braz:** Investigation, Methodology, Writing – original draft, Writing – review & editing.

## Conflicts of interest

The authors declare no conflicts of interest.

## Appendix Supplementary materials

Image, application 1

## Footnotes

Supplementary material associated with this article can be found in the online version at doi:10.1016/j.bjane.2024.844519.
