# Supplementary material for: An update on the mechanisms and risk factors for anesthesia-related cardiac arrest in children: a narrative review
Source: Braz J Anesthesiol. 2024 May 27;74(5):844519. doi: 10.1016/j.bjane.2024.844519 (PMC11252773; doi:10.1016/j.bjane.2024.844519)
Supplement: Supplementary file 1 [file mmc1.docx]

**BJAN-D-23-00559_Supplementary Material**

**Supplementary Table 1** Description of the included studies on anesthesia-related cardiac arrest in children.

| **Investigator and year of publication** | **Data source, country, and study period** | **Median year** | **HDI average** | **Age group anesthesia-related cardiac arrest (n)** | **Age group (n)** | **Exclusions** |
| --- | --- | --- | --- | --- | --- | --- |
| Adekola et al., 2016[24] | Single University Hospital, Retrospective, Nigeria, 2013‒2014 | 2014 | 0.514 | ≤ 18 yr: 5 | ≤ 18 yr: 987 | Cardiac surgery |
| Ahmed et al., 2009[1] | Single University Hospital, Prospective, Pakistan, 1992‒2006 | 1999 | 0.530 | < 18 yr: 4 | < 18 yr: 20,216 | Cardiac surgery |
| Bhananker et al., 2007[20] | Multicenter: 49 University, 11 Community, and 8 Government or Military Hospitals, Retrospective, USA and Canada, 1998‒2004 | 2001 | 0.940 | ≤ 18 yr: 193 | ≤ 18 yr: not related |  |
| Bharti et al., 2009[2] | Tertiary Teaching Pediatric Hospital, Retrospective, India, 2003‒2008 | 2006 | 0.607 | ≤ 17 yr: 9 | ≤ 17 yr: 12,158 | Eye surgery |
|  |  |  |  |  |  | Cardiac surgery |
| Braz et al., 2006[23] | Tertiary University Hospital, Retrospective, Brazil, 1996‒2004 | 2000 | 0.766 | ≤ 17 yr: 7 | ≤ 17 yr: 15,253 |  |
| Bunchungmongkol et al. 2007[25] | Multicenter: 20 Hospitals, Prospective, Thailand, 2003‒2004 | 2004 | 0.784 | ≤ 15 yr: 13 | ≤ 15 yr: 25,098 |  |
| Christensen et al., 2018[14] | Multicenter: 19 University and Private Pediatric Hospitals, Prospective, USA, 2010‒2015 | 2012 | 0.911 | < 18 yr: 329 | < 18 yr: 1,006,609 |  |
| Christensen et al., 2021[38] | University Tertiary Pediatric Hospital, Retrospective, USA, 2006‒2016 | 2011 | 0.940 | < 18 yr: 65 | < 18 yr: 109,775 |  |
| Fiscella et al., 1991[45] | Private Hospital, Prospective, Argentina, 1980‒1990 | 1985 | 0.812 | ≤ 20 yr: 4 | ≤ 20 yr: 2,873 |  |
| Flick et al., 2007[30] | Tertiary University Hospital, Retrospective, USA, 1988‒2005 | 1996 | 0.964 | ≤ 18 yr: 6 | ≤ 18 yr: 92,881 |  |
| Gonzalez et al., 2014[3] | Single Tertiary University Hospital, Retrospective, Brazil, 2005‒2010 | 2008 | 0.750 | ≤ 17 yr: 3 | ≤ 17 yr: 10,649 | . |
| Hohn et al., 2019[21] | Tertiary University Hospital, Retrospective, Germany, 2008‒2016 | 2012 | 0.942 | ≤ 18 yr: 12 | ≤ 18 yr: 36,243 |  |
| Jansen et al., 2021[22] | Tertiary University Hospital, Retrospective, Germany, 2008‒2018 | 2013 | 0.943 | ≤ 15 yr: 9 | ≤ 15 yr: 22,650 |  |
| Kan et al., 2021[51] | Tertiary Hospital, Retrospective, China, 2013‒2020 | 2016 | 0.744 | ≤ 18 yr: 2 | ≤ 18 yr: 17,916 |  |
| Kawashima et al., 2002[46] | Multicenter: 520 Hospitals, Retrospective, Japan, 1999 | 1999 | 0.928 | ≤ 18 yr: 5 | ≤ 18 yr: 115,169 |  |
| Keenan & Boyan, 1985[28] | Tertiary University Hospital, Prospective, USA, 1969‒1983 | 1976 | 0.893 | < 12 yr: 6 | < 12 yr: 12,712 |  |
| Keenan et al.,1991[29] | Tertiary University Hospital, Retrospective, USA, 1983‒1990 | 1987 | 0.941 | ≤ 12 yr: 4 | ≤ 12 yr: 13,893 |  |
| Khoso et al., 2021[44] | Single University Hospital, Retrospective, Pakistan, 1992‒2016 | 2004 | 0.523 | ≤ 16 yr: 13 | ≤ 16 yr: 48,828 | Cardiac surgery |
| Lee et al., 2016[36] | Tertiary University Pediatric Hospital, Retrospective, South Korea, 2008‒2013 | 2010 | 0.914 | ≤ 18 yr: 8 | ≤ 18 yr: 49,373 |  |
| Montobbio et al., 2012[42] | Pediatric Hospital, Prospective, Italy, 2009‒2010 | 2010 | 0.854 | Age group not defined: 2 | Age group not defined: 12,850 |  |
| Morray et al., 2000[19] | Multicenter: 47 University and 25 Pediatric Hospitals, Prospective, USA and Canada, 1994‒1997 | 1996 | 0.935 | ≤ 18 yr: 150 | ≤ 18 yr: 1,089,200 |  |
| Murat et al., 2004[52] | Tertiary University Pediatric Hospital, Prospective, France, 2000‒2002 | 2001 | 0.930 | ≤ 16 yr: 2 | ≤ 16 yr: 23,043 | Cardiac surgery |
|  |  |  |  |  |  | Neurosurgery |
| Newland et al., 2002[43] | Tertiary University Hospital, Prospective, USA, 1989‒1999 | 1994 | 0.948 | ≤ 20 yr: 3 | ≤ 20 yr: 16,051 |  |
| Olsson & Hallén, 1988[40] | Tertiary University Hospital, Retrospective, Sweden, 1967‒1984 | 1976 | 0.934 | ≤ 19 yr: 33 | ≤ 19 yr: 60,827 | Cardiac surgery |
| Rackow et al., 1961[13] | Four Tertiary Hospitals, Retrospective, USA, 1947‒1956 | 1951 | 0.865 | ≤ 12 yr: 19 | ≤ 12 yr: 34,499 |  |
| Ramamoorthy et al., 2010[53] | Multicenter: 49 University, 11 Community, and 8 Government or military hospitals, Retrospective, USA and Canada, 1994‒2005 | 2020 | 0.954 | ≤ 18 yr: 372 | ≤ 18 yr: not related |  |
| Rattana-Arpa et al., 2023[26] | Tertiary University Hospital, Retrospective, Thailand, 2014‒2019 | 2017 | 0.751 | ≤ 17 yr: 34 | ≤ 17 yr: 42,780 | Cardiac surgery |
| Sanabria-Carretero et al., 2013[35] | Quaternary University Pediatric Hospital, Retrospective, Spain, 2007‒2011 | 2009 | 0.900 | ≤ 18 yr: 15 | ≤ 18 yr: 43,291 |  |
| Tiret et al., 1988[41] | Multicenter: 440 University, and Private Hospitals, Prospective, France, 1978‒1982 | 1980 | 0.895 | ≤ 14 yr:12 | ≤ 14 yr: 40,240 |  |
| Zgleszewski et al., 2016[37] | Tertiary University Pediatric Hospital, Prospective, USA, 2000‒2011 | 2005 | 0.938 | ≤ 18 yr: 72 | ≤ 18 yr: 276,209 |  |
| Zoumenou et al., 2010[39] | Two University Hospitals, Retrospective, Benin, 2007 | 2007 | 0.492 | ≤ 15 yr: 8 | ≤ 15 yr: 512 | Cardiothoracic surgery |
|  |  |  |  |  |  | Neurosurgery |

HDI, Human Development Index, ranging from 0 to 1, representing the lowest and highest levels of development, respectively; yr, years.
